# The δ^30^Si peak value discovered in middle Proterozoic chert and its implication for environmental variations in the ancient ocean

**DOI:** 10.1038/srep44000

**Published:** 2017-03-08

**Authors:** T. P. Ding, J. F. Gao, S. H. Tian, C. F. Fan, Y. Zhao, D. F. Wan, J. X. Zhou

**Affiliations:** 1 MLR Key Laboratory of Metallogeny and Mineral Assessment, Institute of Mineral Resources, CAGS, Beijing 100037, P. R. China; 2 Open Research Laboratory of Isotope Geology, CAGS, Beijing 100037, P. R. China

## Abstract

The silicon isotope composition of chert has recently been used to study the historic evolution of the global ocean. It has been suggested that Precambrian cherts have much higher δ^30^Si values than Phanerozoic cherts do and that the former show an increasing trend from 3.5 to 0.85 Ga, reflecting a decrease in ocean temperatures. However, cherts have various origins, and their isotopic compositions might be reset by metamorphic fluid circulation; thus, different types of cherts should be distinguished. Here, we present a new set of δ^30^Si data for cherts from early and middle Proterozoic carbonate rocks from Northern China. We found that cherts of 1.355–1.325 Ga show a peak range of 2.2–3.9‰. Based on these results, we propose that from the Archean to the middle Proterozoic, there was a drastic decrease in silicon content and an increase in the δ^30^Si value in ocean water due to a temperature decrease and biological activity increase. After that period, the silicon content of the ocean was limited to a low level by a high degree of biological absorption, and their δ^30^Si values varied in a small range around a significantly lower value.

Chert is a sedimentary rock composed mainly of microcrystalline quartz. It occurs in sedimentary strata from the early Archean to the present. Its chemical and isotopic characteristics have been widely investigated to study the conditions of its formation. Recently, Si isotopes in chert have been used as an important tracer of environmental conditions in the global ocean^1^,^2^,^3^,^4^,^5^,^6^,^7^,^8^,^9^,^10^,^11^,^12^,^13^,^14^,^15^,^16^,^17^,^18^. Song and Ding (1990) suggested distinguishing sedimentary facies of chert with silicon isotope compositions^1^. Ding *et al*. (1996) indicated that different genetic types of cherts have different silicon isotope characters^2^. They discussed variations in silicon content and silicon isotope composition in marine water according to the data from chert formed in shallow marine environments. According to the silicon isotope variation of chert, Robert and Chaussidon (2006) developed a temperature-evolution curve for the Precambrian ocean^3^. It has been suggested that Precambrian cherts have much higher δ^30^Si values than Phanerozoic cherts and that the former show a generally increasing trend from 3.5 to 0.85 Ga, thus reflecting a decrease in seawater temperature^3^. However, these statements have been challenged because cherts can have various origins and their isotopic compositions might have been reset by metamorphic fluid circulation^4^,^5^,^6^,^7^,^8^,^9^. Thus, different types of cherts need to be considered separately^4^,^9^,^10^,^11^,^12^ because their isotope composition might record various distinct processes^13^,^14^,^15^,^16^,^17^,^18^. In addition, the factors that affect silicon isotope composition of chert have been investigated in detail^13^,^14^,^15^,^16^,^17^,^18^. It was found that peritidal cherts are enriched in ^30^Si, but that basinal cherts, which are associated with banded iron formations (BIF), are depleted in ^30^Si; this difference is attributed to Si having been derived from hydrothermal sources in the BIFs^10^. Now, it is widely accepted that the formation of chert is a complicated process and that the Si isotope composition of chert is dependent on the relative contributions of various Si sources and the effects of different forming processes^9^,^10^,^11^,^12^,^13^,^14^,^15^,^16^,^17^,^18^. However, the relative contributions of the various Si sources to the global ocean vary throughout geological history. Thus, specific types of chert might be representative for reconstructing the Si isotope composition of the ocean for different geological periods. For example, during the Archean period, sea-floor weathering and submarine hydrothermal fluids dominated the Si input to the ocean, and continent inputs were negligible; thus, the Si isotope compositions of quartz bands in BIF are likely the best approach for tracing the Si isotope compositions of the ocean. In contrast, in the Proterozoic and Phanerozoic periods, the Si input from the continent to the ocean became dominant, and the Si input from sea-floor weathering and submarine hydrothermal fluids became less prevalent; thus, peritidal chert may be more significant in tracing the Si isotope composition of the ocean. To date, many studies have investigated the silicon isotope composition of silica in BIF to examine the environmental conditions of the Archean ocean^2^,^3^,^4^,^5^,^6^,^7^,^8^,^9^,^11^,^12^,^13^,^14^. However, studies specifically targeting the silicon isotope composition of cherts formed in shallow marine environment are relatively rare^10^. More systematic and detailed Si isotope studies on this aspect must be undertaken to reconstruct the silicon content and silicon isotope composition in the ancient ocean and understand the historic evolution of the global ocean.

We collected a suite of samples of carbonate rocks containing chert bands and nodules from the early Proterozoic Hutuo Group^19^ (2.35–2.20 Ga) and the middle Proterozoic Changcheng and Jixian Systems^20^,^21^ (1.63–1.20 Ga) in Northern China (Fig. 1). These cherts were selected because they showed good preservation of their original structure and composition (Figs 2 and 3); i.e., no obvious effects of metamorphism or weathering were found in the selected samples. The Si and O isotopic compositions of the chert and the C and O isotopic compositions of dolomite were studied systematically.

## Results

The Si and O isotope compositions of the cherts and the C and O isotope compositions of dolomites obtained in this study are listed in Table 1 and shown in Fig. 4. All isotope compositions are reported in delta-notation relative to a standard material, i.e.,


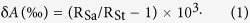


where A represents the isotope, R represents the isotope ratio, the subscript Sa refers to the sample and St refers to the standard. For the Si isotope compositions, A means ^30^Si, R means ^30^Si/^28^Si, St means NBS-28. For the O isotope composition, A means ^18^O, R means ^18^O/^16^O, and St can be either V-SMOW (for chert and dolomite) or V-PDB (for dolomite). For the C isotope composition, A means ^13^C, R means ^13^C/^12^C, and St means V-PDB.

### The δ^13^C _V-PDB_ and δ^18^O_V-PDB_ values of dolomites

The obtained δ^13^C_V-PDB_ values of dolomites range from −2.5‰ to 1.9‰, with an average of −0.36 ± 0.82 (1 SD) ‰. The obtained δ^18^O_V-PDB_ values of dolomites range from −12.9‰ to −3.5‰, with an average of −7.11 ± 2.15 (1 SD) ‰. These values are similar to those reported in previous studies^22^,^23^, reflecting their normal sedimentary origin in a shallow marine environment. The δ^18^O_V-PDB_ values indicate that the dolomite had been influenced to some degree by diagenesis.

### The δ^18^O_V-SMOW_ values of cherts

The δ^18^O_V-SMOW_ values of the chert samples range from 14.3‰ to 27.8‰, with an average of 23.0 ± 3.5 (1 SD) ‰. These results also show that they were formed in a shallow marine environment but might have been slightly affected by diagenesis.

### The δ^30^Si_NBS-28_ values of cherts

The δ^30^Si values of the cherts collected in this study range from 0.1‰ to 3.9‰. Among them, the early Proterozoic cherts show lower δ^30^Si values, ranging from 0.1‰ to 1.3‰, with an average of 0.75 ± 0.43‰ (1 SD), compared to middle Proterozoic cherts ranging from 0.5‰ to 3.9‰, with an average of 2.22 ± 0.74‰ (1 SD). Furthermore, a peak range in δ^30^Si [2.2–3.9‰, averaging 3.12 ± 0.56‰ (1 SD)] is observed in the cherts of 1.325–1.355 Ga (Fig. 4). In addition, the δ^30^Si variation of chert can also be observed on a millimetre scale. For example, 4 chert bands in sample JX-26 and 5 chert bands in sample JX-27 show δ^30^Si variations of 2.3∼3.9‰ and 3.1∼3.6‰, respectively (Table 1).

## Discussion

The increasing trend of δ^30^Si from the early Proterozoic to middle Proterozoic is consistent with the trends reported by previous studies^2^,^3^,^5^,^6^,^7^,^8^,^9^,^10^. However, together with the new data provided in this study, the middle Proterozoic δ^30^Si peak becomes a prominent feature in the Si isotope record of Precambrian cherts, which implies major environmental variations in the ancient ocean (Fig. 4).

Combining data obtained using SiF_4_ and MC-ICP-MS methods in this study and previous studies^10^,^24^,^25^,^26^,^27^,^28^,^29^,^30^, the δ^30^Si variation trend from the late Archean to present is plotted for chert that formed in shallow marine environments (Fig. 5). The upper limit of δ^30^Si values of chert increases gradually from 1.8‰ at 2.53 Ga to 3.9‰ at 1.335 Ga and then decreases drastically to 2.0‰ at 1.104 Ga. After 1.104 Ga, the upper limit of δ^30^Si values for chert fluctuates between 1.5‰ and 2.5‰.

Chert is normally formed by the recrystallization of a precipitated amorphous silica precursor in the diagenetic process. To test the possibility of using O and Si isotope compositions of chert to trace those of contemporary marine water, the relationship between the δ^18^O and δ^30^Si values of chert and those of the amorphous silica precursor must be evaluated first.

**Figure 3:**
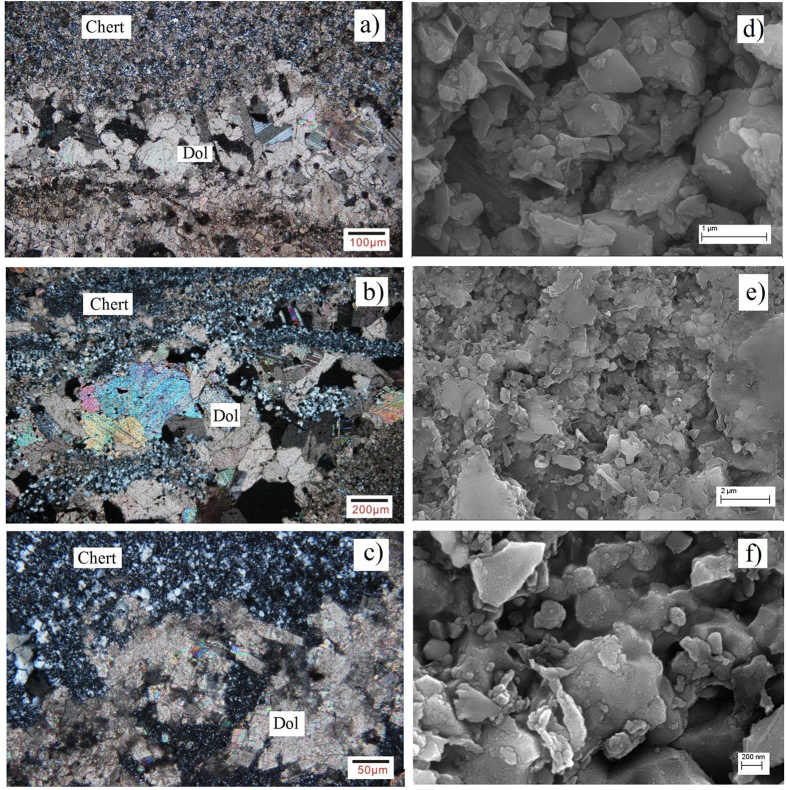
Microscopic and SEM photos of chert and dolomite. **a**), **b**) and **c**) are microscopic photos (crossed Nicols) of samples JX-26, JX-27 and JX-33, respectively; **d**), **e**) and **f**) are SEM images of samples JX-26, JX-27 and JX-27, respectively. Generally the chert consists of microscopic quartz grains ranging from 0.1 μm to a few μm in diameter. In some samples (JX-33), coarser quartz crystals of >10 μm can be observed. The chert seems to have re-crystallized after the dolomite, indicating a diagenetic origin.

**Figure 4:**
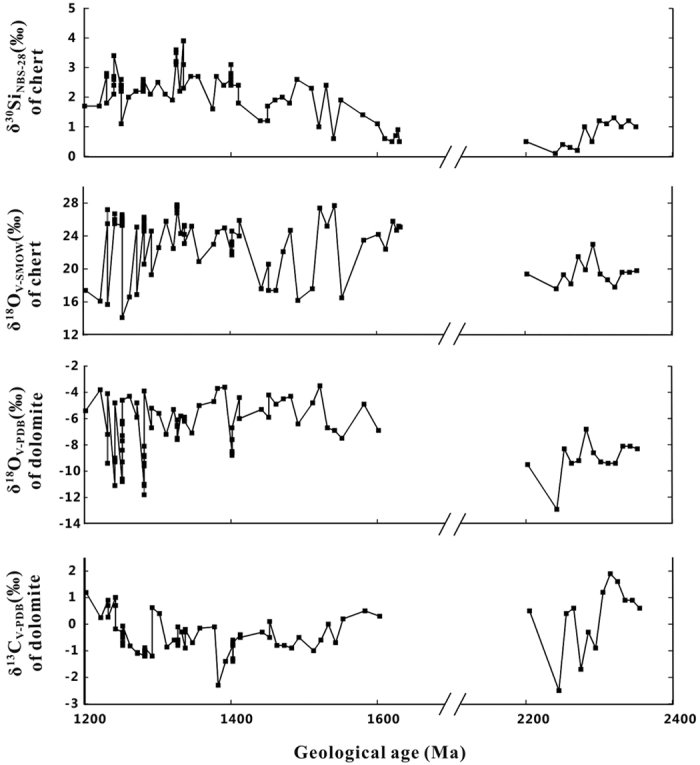
The temporal evolution of δ^30^Si_NBS-28_ and δ^18^O_V-SMOW_ values of chert and δ^13^C _V-PDB_ and δ^18^O_V-PDB_ values of dolomite for the samples collected in this study. Analytical precisions are provided in Table 1.

It is known that the O isotope composition of silica would be reduced during diagenesis due to two factors. First, the chert nodular and band in the limestone are considered to form in the groundwater of mixed meteoric-marine coastal systems during diagenesis^31^. Due to the involvement of meteoric water, the O isotope composition of groundwater would be lighter than that of contemporary marine water, causing a reduction in the δ^18^O value of chert to some extent. Second, as the diagenetic temperature is normally higher than that of marine water, the O isotope fractionation between silica and water at the diagenetic stage would be smaller than that in the precipitation stage, which would cause a δ^18^O reduction in the chert. As shown by the microscopic and SEM examinations (Fig. 3), the slight reduction in the δ^18^O value of chert indicates that the studied cherts were formed in the diagenetic process and their δ^18^O values cannot represent those of the amorphous silica precursor.

In contrast to the O isotope composition, the silicon isotope composition would not change during early diagenesis for following reasons: (1) Chert bands and nodules are commonly confined in layers of sedimentary strata, which indicate that silica does not move over long distances during diagenesis. (2) The amorphous silica precursor is the dominant form of silica in dolomite rocks and the Si content in groundwater is rather limited. (3) δ^30^Si variations at the millimetre scale or even the micrometre scale^3^,^8^,^13^,^14^,^32^,^33^ are preserved in cherts, which rules out the possibility of the large-scale mixing of silicon during diagenesis. Thus, the δ^30^Si value of chert can be used as a representative of the amorphous silica precursor to trace the silicon isotope composition of contemporary seawater^5^.

**Figure 5:**
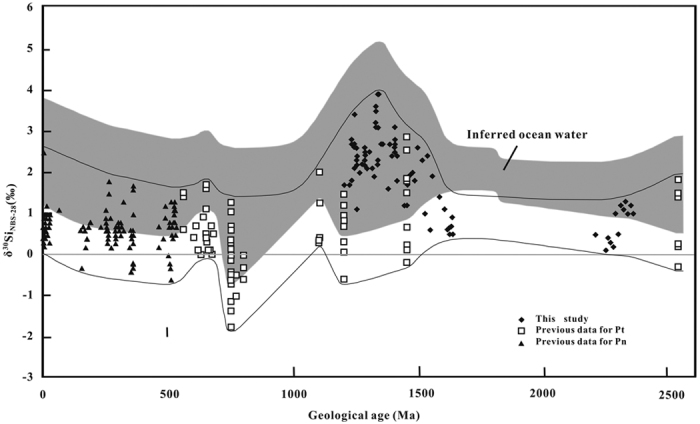
A plot showing δ^30^Si variations of chert formed in a shallow marine environment, and inferred ocean water, from the late Archean to the present. Previous data for Proterozoic chert (Pt)^10^,^24^,^25^,^26^ and Phanerozoic chert (Pn)^2^,^24^,^27^,^28^,^29^,^30^ are included. Taking Δ^30^Si_Ch-SW_ to be −0.5‰, −1.0‰ and −1.2‰ in the Archean, early Proterozoic and since the middle Proterozoic, respectively, δ30SiSW values are calculated from δ^30^Si_Ch_.

In Fig. 5, the inferred variation trend in δ^30^Si_SW_ (δ^30^Si value of seawater) is shown. The δ^30^Si_SW_ is calculated from the equation δ^30^Si_SW_ = δ^30^Si_Ch_ − Δ^30^Si_Ch-SW_, where δ^30^Si_Ch_ is the δ^30^Si value of chert and Δ^30^Si_Ch-SW_ is the relative Si isotope enrichment of chert to seawater. According to the theory of isotope fractionation, Δ^30^Si_Ch-SW_ is temperature dependent. Thus, to determine a proper Δ^30^Si_Ch-SW_ value, precipitation temperatures of the amorphous silica precursor of chert should be evaluated beforehand.

Based on the O isotope composition of chert, Robert and Chaussidon (2006)^3^ suggested the seawater temperatures were 70 °C at 3.5 Ga and 35 °C at 0.85 Ga. However, the inferred high Paleoarchean temperatures are controversial^34^,^35^,^36^,^37^,^38^, partly because δ^18^O determinations of the Paleoarchean temperature rely on the assumption that δ^18^O of the Archean ocean was similar to that of an ice-free modern ocean. However, it has been suggested by a number of researchers that the δ^18^O value of the global ocean could have varied significantly over time^35^,^36^,^37^,^38^. Recently Hren *et al*.^34^ studied the δ^18^O and δD values of the 3.42 Ga Buck Reef Chert rocks in South Africa and found that the chert with the highest δ^18^O was formed in equilibrium with waters below 40 °C. Blake *et al*. (2010) studied the oxygen isotope composition of phosphate in 3.2–3.5 Ga sediments of the Barberton Greenstone Belt and found that the phosphate with the highest δ^18^O value was formed in seawater at a temperature range from 26 °C to 35 °C^39^. According to these results, a temperature range of 35 °C–40 °C is more acceptable for the Archean ocean.

For the temperature of the Proterozoic ocean, fewer results have been reported. Based on the O isotope composition of chert, the temperatures in the Proterozoic ocean are estimated in the range of 35–60 °C^3^. According to the δ^18^O values of chert and dolomite obtained in this study, the diagenetic temperature can be estimated. Assuming that diagenetic water has a δ^18^O value of −10‰, and using the O isotope fractionation equation (1000lnα_Dol-H2O_ = 3.20 × 10^6^T^−2^−2.0) of Northrop and Clayton (1966)^40^, we obtained a diagenetic temperature range of 25~56 °C (averaging 36 ± 7 °C) for dolomite in the early Proterozoic and a diagenetic temperature range of 12~50 °C (averaging 26 ± 9 °C) for dolomite in the middle Proterozoic. Assuming the diagenetic water still has a δ^18^O value of −10‰, and using the O isotope fractionation equation (1000lnα_Chert-H2O_ = 3.09 × 10^6^T^−2^−3.29) of Knauth & Epstein (1975)^41^, we obtained a diagenetic temperature range of 19~43 °C (averaging 34 ± 7 °C) for chert in the early Proterozoic and 1~63 °C (averaging 17 ± 15 °C) for chert in the middle Proterozoic. The calculated average diagenetic temperature (34 °C) for early Proterozoic chert is slightly lower than that (36 °C) for early Proterozoic dolomite, and the calculated average diagenetic temperature (17 °C) for middle Proterozoic chert is significantly lower than that (26 °C) for middle Proterozoic dolomite. These observations may be caused by a difference between chert and dolomite during O isotope exchange process with diagenetic solution. The cherts are more resistant to O isotope exchange than dolomite in the diagenetic process. Thus, the calculated diagenetic temperature for dolomite may be more representative. Based on these considerations and assuming the diagenetic temperature is a little higher than the sedimentary temperature on the sea floor, we estimate the ocean temperature as 30 °C in the early Proterozoic and 20 °C in the middle Proterozoic.

Concerning Δ^30^Si_Ch-SW_, there has been a number of investigations on Si isotope fractionation during abiotic silica precipitation^15^,^16^,^17^,^42^,^43^,^44^,^45^. Early experimental studies on abiotic solid–fluid silicon isotope fractionation yielded Δ^30^Si_solid–fluid_ values ranging from −2.0‰ to −1.0‰^42^,^44^. Geilert *et al*. (2014) performed seeded silica precipitation experiments using flow-through reactors in the 10–60 °C temperature range to quantify the silicon isotope fractionations during controlled precipitation of amorphous silica from a flowing aqueous solution^15^. The obtained Δ^30^Si_Silica-solution_ values were −2.1‰ at 10 °C, −1.2‰ at 20 °C, −1.0‰ at 30 °C, −0.5‰ at 40 °C, 0.1‰ at 50 °C, and 0.2‰ at 60 °C. These results can be used to calculate δ^30^Si values of ocean water in different geological periods.

Assuming that the temperature of the ocean is 40 °C, 30 °C and 20 °C in the Archean, early Proterozoic and since the middle Proterozoic, respectively, and Δ^30^Si_Ch-SW_ are −0.5‰, −1.0‰ and −1.2‰ in the Archean, early Proterozoic and since the middle Proterozoic, respectively, δ^30^Si values of ocean water are calculated from δ^30^Si values of chert (Fig. 5).

Figure 5 shows that the upper limit of inferred δ^30^Si_NBS-28_ values in ocean water increases gradually from 2.8‰ at 2.53 Ga to 5.1‰ at 1.335 Ga and then decreases drastically to 3.2‰ at 1.104 Ga. After 1.104 Ga, the upper limit of inferred δ^30^Si_NBS-28_ values in ocean water fluctuates between 2.7‰ and 3.7‰.

**Figure 6:**
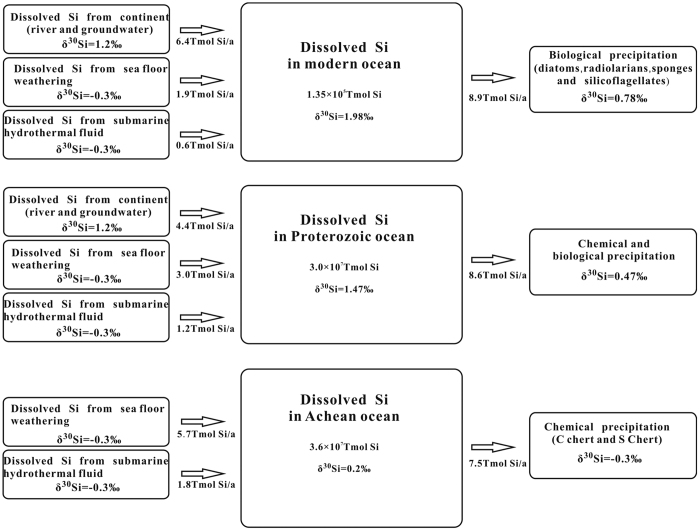
A schematic diagram showing Si cycles in the Archean^4^,^5^,^6^, Proterozoic and modern oceans^29^.

As shown in Fig. 6, during the Archean period, the input sources of dissolved Si to the ocean are submarine hydrothermal fluid and sea-floor weathering, and the output paths are chemical precipitation (to form C cherts) and silicification of the precursor sediments or rocks (to form S cherts)^4^,^5^,^6^. The Si concentration in ocean water remains in its saturated concentration at a given temperature, but the δ^30^Si_SW_ increases gradually due to Si isotope fractionation between dissolved Si and precipitated SiO_2_. When a steady state is reached, δ^30^Si_Out_ (the average δ^30^Si value of all output Si) will be equivalent to δ^30^Si_In_ (the average δ^30^Si value of input Si), and δ^30^Si_SW_ will be equal to (δ^30^Si_Out_ -Δ^30^Si_Out -SW_), where Δ^30^Si_Out -SW_ is the relative silicon isotope enrichment of the output Si to the ocean water (δ^30^Si_Out_ - δ^30^Si_SW_). Because the average δ^30^Si value of Si in the submarine hydrothermal fluid and Si from sea-floor weathering is ~−0.3‰ and Δ^30^Si_Out-SW_ is ~−0.5‰ at a temperature of 40 °C^15^, the δ^30^Si_SW_ value of that period is approximately 0.2‰.

The Si cycle in the modern ocean (Fig. 6) is quite different^46^. First, dissolved Si from the continents (in rivers^47^ and groundwater) has become a dominant input source (6.4 Tmol Si/a) to ocean Si, and the Si input from submarine hydrothermal fluid (0.6 Tmol Si/a) and sea-floor weathering (1.9 Tmol Si/a) has become less significant^46^. δ^30^Si_in_ is calculated as ~0.78‰ using the equation


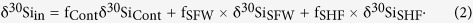


In the equation, f represents the relative fraction of each Si source and the subscripts Cont, SFW and SHF indicate continent, sea-floor weathering and submarine hydrothermal fluid, respectively.

Second, the biological absorption of Si has become a dominant path for Si output from the ocean and Si contents in modern ocean water (0.05 mg/L~0.2 mg/L for shallow seawater and 0.3 mg/L~3.5 mg/L for deep seawater) are 2 orders of magnitude lower than those of the saturated concentration in seawater^2^. At steady state, the amount and δ^30^Si value of output Si from ocean water would be equal to those of input Si. Thus, δ^30^Si_Out_ would also be ~0.78‰ at present.

The silicon isotope fractionations of diatoms-seawater and sponges-seawater have been experimentally studied. The determined Δ^30^Si_Diatom-SW_ is commonly −1.0∼−1.1‰^48^,^49^,^50^, but Δ^30^Si_Sponge-SW_ varies from −1.1‰ to −3.7‰^51^,^52^. Because diatoms are much more abundant in the ocean than sponges, we assume Δ^30^Si_Out-SW_ in the modern ocean is ~−1.2‰. From the above estimation, the δ^30^Si_SW_ value of the modern ocean can be calculated as ~1.98‰, which is very close to the value (1.9‰) of surface ocean water inferred previously^53^.

Many papers have reported the δ^30^Si values of cherts formed during the Proterozoic^1^,^2^,^3^,^8^,^9^,^10^,^24^,^25^,^26^,^32^, but no model of the Si cycle in the Proterozoic ocean has been presented. Here, we present a conceptual model for the Si cycle of the Proterozoic ocean based on known and inferred boundary conditions (Fig. 6). Similar to the conditions in the Archean ocean, submarine hydrothermal fluid and sea-floor weathering are still important input sources of dissolved Si to the Proterozoic ocean, but the amounts of these inputs decreased as the hydrothermal activity and ocean temperature decreased from their Archean to Proterozoic values. Further, the input of dissolved Si from the continents became significant as supercontinents appeared in the early Proterozoic. For the output of dissolved Si from the ocean in the Proterozoic Eon^54^, chemical precipitation was still a major pathway, but biological absorption may have also played a significant role.

The Si concentration in ocean water remains in its saturated concentration at a given temperature (30 °C for the early Proterozoic and 20 °C for the middle and late Proterozoic). When a steady state is reached, δ^30^Si_Out_ will be equivalent to δ^30^Si_In_, and δ^30^Si_SW_ will be equal to (δ^30^Si_Out_ − Δ^30^Si_Out -SW_), where Δ^30^Si_Out -SW_ is −1.0‰ for the early Proterozoic and −1.2‰ for the middle and late Proterozoic. The estimated δ^30^Si_SW_ value of that period would be approximately 1.47‰ (Fig. 6).

From the discussion above, extreme values of δ^30^Si for chert and δ^30^Si for seawater cannot be explained for an ocean at steady state conditions. Thus, this peak in δ^30^Si indicates an extraordinary period at non-steady state suggesting a scenario at a transition stage.

**Figure 7:**
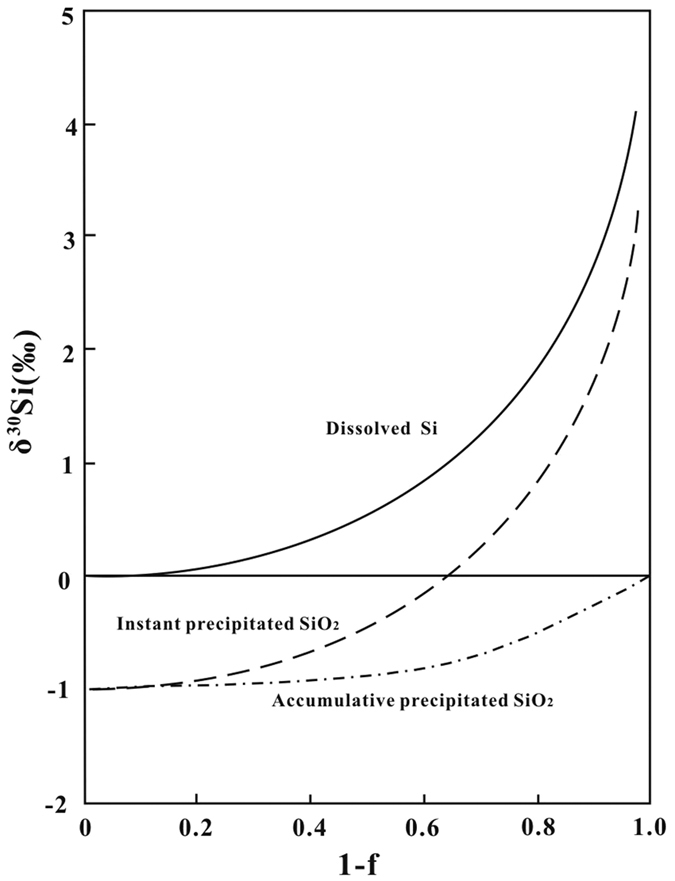
Plot showing silicon isotope fractionation during SiO_2_ precipitation from ocean water in a Rayleigh process, where f is the fraction of remaining dissolved Si in ocean water and 1-f is the fraction of accumulated precipitated SiO_2_. The δ^30^Si of the starting ocean water is assumed to be 0‰ and the Si isotope fractionation factor between SiO_2_ precipitate and ocean water (α_Pre-SW_) is 0.999.

From the Archean to present, there should be a transition period of the Si cycle in the ocean. In that period, the D_Si_ in ocean water is reduced by approximately 2 orders of magnitude below that of the saturated concentration in seawater^2^, and the δ^30^Si_SW_ value first rises from ~0.2‰ to ~5.1‰ and then decreases to ~1.98‰. The rise of δ^30^Si_SW_ is caused by Rayleigh fractionation when SiO_2_ precipitates from ocean water (Fig. 7). One mechanism that causes the D_Si_ reduction in ocean water should be a decrease in ocean temperature. As temperature decreases, the saturated Si concentration in ocean water would be reduced, causing additional SiO_2_ precipitation. The saturated SiO_2_ concentration in ocean water is ~363.5 mg/L, ~221.2 mg/L and ~178.9 mg/L at 40 °C, 30 °C and 20 °C, respectively^55^. When the temperature of seawater decreases from 40 °C to 20 °C, the fraction of dissolved Si remaining in the seawater (f) will be reduced to ~0.687. In the Rayleigh fractionation process, it will cause an increase of ~0.2‰ in δ^30^Si_SW_. It seems that the decrease in seawater temperature alone cannot explain the significant increase in the δ^30^Si_SW_ value, and other mechanisms should be considered. Another mechanism causing a D_Si_ decrease in ocean water is the increase of Si absorption activities by biological species, which can reduce the D_Si_ in ocean water 2 orders of magnitude lower than that of the saturated concentration. In the Rayleigh fractionation process, the combined effect of these two types of mechanisms can cause δ^30^Si_SW_ to increase ~4.0‰ when f is reduced to 0.01. It is known that diatoms and radiolarians are Si-fixing organisms that were active in the Phanerozoic^46^; sponges were active in the Phanerozoic^46^ and the later Proterozoic^54^. Assuming their appearance is the start of a drastic decrease of D_Si_ in ocean water, we should observe a δ^30^Si_SW_ peak value in the later Proterozoic or early Phanerozoic. However, according to the data here, the δ^30^Si_SW_ peak appeared in the middle Proterozoic (1.325~1.355 Ga) instead, which indicates the drastic decrease in ocean water D_Si_ happened prior to 1.355 Ga.

It is known that microbes were the dominant biological species in the Precambrian. Stromatolites are found in early Archean strata from 3.5 Ga^56^ and are very well developed in Proterozoic strata^19^,^20^,^21^,^22^,^23^,^57^. REE and C, O, Nd isotope compositions have been used to study the formation conditions of stromatolite-bearing sediments, particularly the effect of biological activities^22^,^23^,^56^,^57^,^58^. The early and middle Proterozoic chert-bearing dolomites investigated in this study are all rich in stromatolites, showing a close correlation between silica precipitation and biological activities. Moreover, macroscopic eukaryotic fossils were recently discovered in the 1.56 Ga Gaoyuzhuang Formation in the Yanshan area of Northern China^59^. If some Proterozoic species are capable of absorbing or precipitating Si from ocean water, the Si content in the Proterozoic ocean water would be drastically reduced causing δ^30^Si_SW_ to rise significantly. Thus, the high peak in δ^30^Si_SW_ values in the middle Proterozoic ocean water may reflect a drastic reduction in Si content caused by a rapid increase in biological activity in the ocean. After that peak period, the D_Si_ reduction rate in ocean water decreased gradually, and the δ^30^Si_SW_ value decreases to a significantly lower value at steady state.

## Methods

### Si and O isotope analysis of chert samples

For Si and O isotope analysis, ~100 mg of a chert band or nodule was selected from a polished section of each specimen. The sample was crushed and ground to a powder of −200 mesh. Then, the sample powder was reacted with 6 N HCl in Teflon beakers to dissolve small amounts of carbonate. The remainder was washed at least in triplicate with Milli-Q water. Then, the remainder was transferred to a Pt crucible, dried at 105 °C in an oven and then calcined at 1000°C in a muffle furnace to remove organic C impurities.

Oxygen isotope analyses were carried out using the BrF_5_ method (Clayton and Mayeda, 1963)^60^, and silicon isotope analyses were carried out using the SiF_4_ method (Ding, 2004)^61^. Approximately 10 mg of pretreated chert was placed in a Ni reactor in a metal vacuum line and reacted with BrF_5_ at a temperature of approximately 500°C to produce gaseous O_2_ and SiF_4_.

The O_2_ gas was separated from SiF_4_, BrF_5_ and BrF_3_ by evaporating at liquid nitrogen temperature. Then, O_2_ gas was converted to CO_2_ by reacting with a carbon rod at 700 °C. Finally, the CO_2_ gas was collected for O_2_ isotope measurement.

SiF_4_ was separated from the BrF_5_ and BrF_3_ by evaporating at dry ice-acetone temperature. The separated SiF_4_ was purified further by passing it through a Cu tube containing pure Zn particles at a temperature of 60°C. This procedure removed trace amounts of the remaining active F-bearing compounds (BrF_5_ and BrF_3_). Then, the purified SiF_4_ was collected for silicon isotope measurement.

The isotopic measurements were carried out with a MAT-253 mass spectrometer.

For O_2_ isotope measurement, the NIST Standard Reference Material for O isotopes (NBS-28) was used directly as the working standard in this study. The precision of the O isotope measurement is better than ± 0.2‰ (2σ). The O isotope compositions of all samples are reported as δ^18^O values relative to the V-SMOW standard.

For Si isotope measurements, international reference material for Si isotopes (NBS-28) and two Chinese national standards for Si isotopes (GBW04421 and 04422) were used as working standards in this study. The long-term reproducibility of the silicon isotope measurements is better than ± 0.1‰ (2σ). The silicon isotope compositions of all samples are reported as δ^30^Si values relative to the NBS-28 standard.

### C and O isotope analysis of dolomite samples

The continuous-flow isotope-ratio mass spectrometric method was used for C and O isotope analysis of dolomite^62^. The system consists of a Thermo-Finnigan GasBench II equipped with a CTC Combi-Pal auto-sampler and linked to a Finnigan MAT 253 mass spectrometer.

Approximately 100 mg of dolomite was taken from the specimen and ground to a powder of −200 mesh. Approximately 100 μg of dolomite powder was loaded manually into a 12 ml round-bottomed borosilicate exetainer and sealed using butyl rubber septa. Four national reference materials (GBW04405, GBW04406, GBW04416 and GBW04417) were routinely loaded. The exetainers were automatically flushed with grade 5 He by penetrating the septa using a double-hole needle at a flow rate of 100 mL/min. Then, 4–6 drops of phosphoric acid were deposited in each exetainer. The exetainers were placed onto an aluminium tray and kept at 72 °C for 24 h. Subsequently, the sample gas was introduced into the mass spectrometer through the standard 100 μL sample loop, CO_2_ was separated from other components using a gas chromatographic column heated to 70 °C, and the peak corresponding to this CO_2_ was passed via an open split to the mass spectrometer.

The calculated external precision is typically ± 0.2‰ (2σ) for δ^13^C and δ^18^O. The C isotopic compositions of the dolomite samples are reported as δ^13^C values relative to the V-PDB standard. The O isotopic compositions of the dolomite samples are reported as δ^18^O values relative to V-PDB and V-SMOW standards.

## Additional Information

**How to cite this article**: Ding, T. P. *et al*. The δ^30^Si peak value discovered in middle Proterozoic chert and its implication for environmental variations in the ancient ocean. *Sci. Rep.* **7**, 44000; doi: 10.1038/srep44000 (2017).

**Publisher's note:** Springer Nature remains neutral with regard to jurisdictional claims in published maps and institutional affiliations.

## Figures and Tables

**Figure 1:**
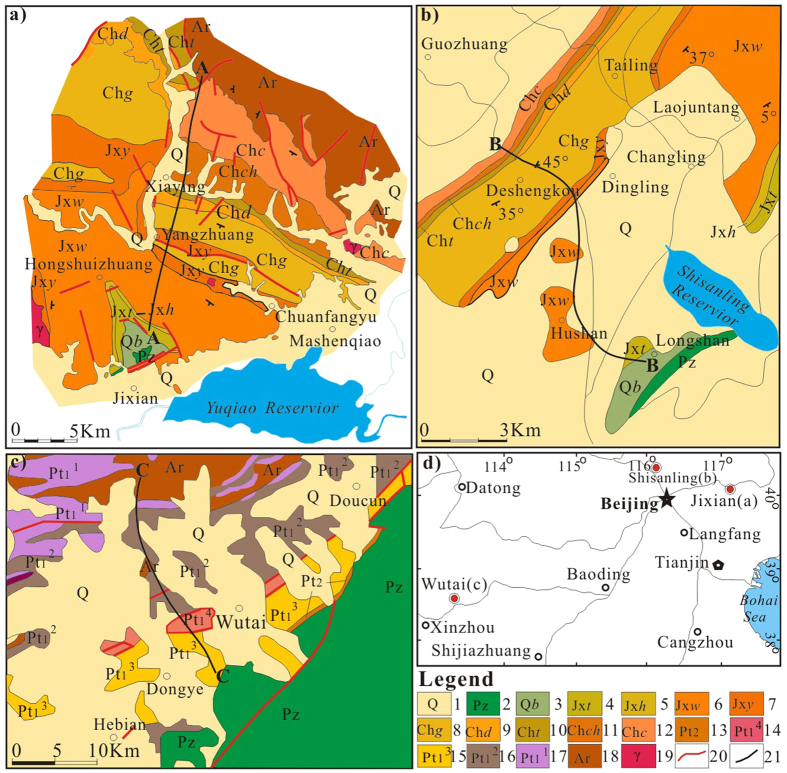
Schematic geological maps of sampling area. (**a**) Map of the Jixian area, Hebei province^20^; (**b**) Map of the Shisanlin area, Beijing^21^; (**c**) Map of the Wutai area, Shanxi province^19^; (**d**) A sketch map showing the locations of sampling areas. The sampling areas of Jixian, Shisanlin and Wutai are indicated by red solid cycles in the map. 1-Quaternary strata; 2-Phanerozoic strata; 3-Upper Proterozoic Qingbaikou System; 4-Tieling Fm., Jixian system; 5-Hongshuizhuang Fm., Jixian System; 6-Wumishan Fm., Jixian System; 7-Yangzhuang Fm., Jixian System; 8-Gaoyuzhuang Fm., Changcheng System; 9-Dahongyu Fm., Changcheng System; 10-Tuanshanzi Fm., Changcheng System; 11-Chuanlinggou Fm., Changcheng System; 12-Changzougou Fm., Changcheng System; 13-Middle Proterozoic strata; 14-Sijizhuang Fm., Hutuo Group; 15-Doucun Fm., Hutuo Group; 16-Dongye Fm., Hutuo Group; 17-Guojiazai Fm., Hutuo Group; 18- later Archean Wutai strata; 19-Granite; 20-Fault; 21- Sampling profiles in Jixian (**a**, A-A), Shisanlin (**b**, B-B) and Wutai (**c**, C-C). The software CorelDRAW X3 (13.0.0.667) was used to create the maps. The URL is http://www.corel.com.

**Figure 2:**
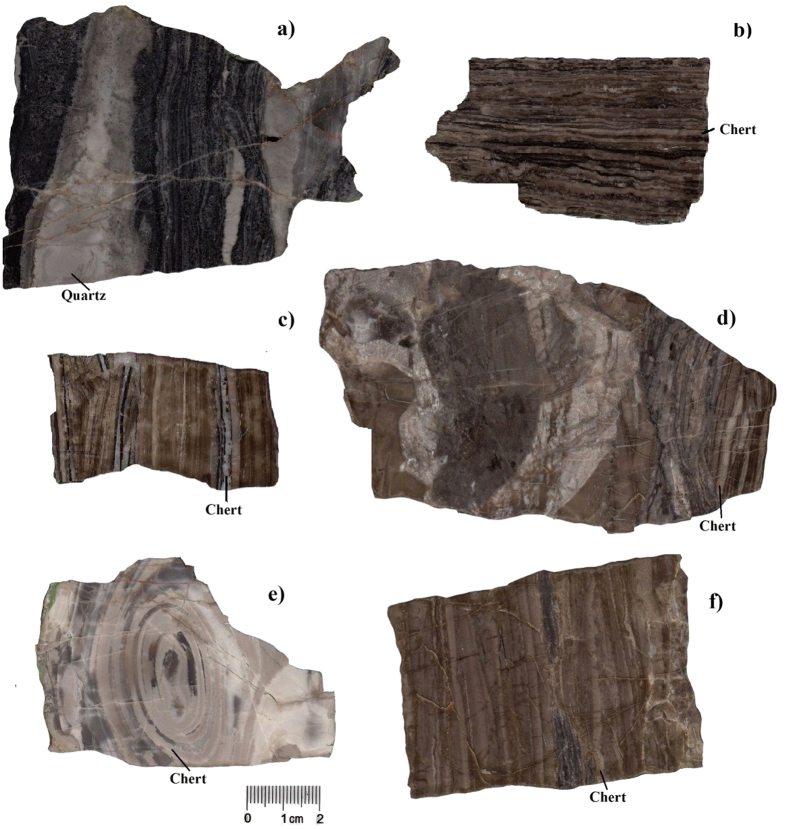
Photos of polished sections of sample specimens. (**a**) JX-20; (**b**) JX-26; (**c**) JX-27; (**d**) JX-33; (**e**) JX-36; (**f**) JX-40. The cherts commonly occur as fine bands, but quartz veins can be observed in some specimens (JX-20, JX-33 and JX-36).

**Table 1:** δ^30^Si_NBS-28_ and δ^18^O_V-SMOW_ values for chert and δ^13^C _V-PDB_ and δ^18^O_V-PDB_ values for dolomite for the early Proterozoic Hutuo Group and middle Proterozoic Changcheng and Jixian Systems.

Sample No.	Sample type	Strata	Age (Ma)	CaMg (CO_3_)_2_			SiO_2_	
				δ^13^C_V-PDB_ (‰)	δ^18^O_V-PDB_ (‰)	δ^18^O_V-SMOW_ (‰)	δ^18^O_V-SMOW_ (‰)	δ^30^Si_NBS-28_ (‰)
Hutuo Group in Wutai profile								
HT-03	Purple-red fine banded cherty Dol.	Hebiancun Fm., Hutuo Gr.	2350	0.6	−8.3	22.4	19.8	1.0
HT-04	Purple-red cherty Dol.	Hebiancun Fm., Hutuo Gr.	2340	0.9	−8.1	22.6	19.6	1.2
HT-05	Purple-red fine banded cherty Dol.	Hebiancun Fm., Hutuo Gr.	2330	0.9	−8.1	22.6	19.6	1.0
HT-06	Yellow fine banded cherty Dol.	Hebiancun Fm., Hutuo Gr.	2320	1.6	−9.4	21.2	17.8	1.3
HT-07	Purple-red fine banded cherty Dol.	Hebiancun Fm., Hutuo Gr.	2310	1.9	−9.4	21.2	18.7	1.1
HT-08	Grey banded cherty Dol.	Hebiancun Fm., Hutuo Gr.	2300	1.2	−9.3	21.3	19.4	1.2
HT-09	Dark grey fine banded cherty Dol.	Hebiancun Fm., Hutuo Gr.	2290	−0.9	−8.6	22.0	23.0	0.5
HT-10	Purple-red cherty Dol.	Hebiancun Fm., Hutuo Gr.	2280	−0.3	−6.8	23.9	19.9	1.0
HT-11	Dark grey cherty Dol.	Hebiancun Fm., Hutuo Gr.	2270	−1.7	−9.2	21.4	21.5	0.2
HT-12	Grey fine banded cherty Dol.	Hebiancun Fm., Hutuo Gr.	2260	0.6	−9.4	21.2	18.2	0.3
HT-14	Grey banded cherty Dol.	Jianan Fm., Hutuo Gr,	2250	0.4	−8.3	22.3	19.3	0.4
HT-15	Dark grey cherty Dol.	Jianan Fm., Hutuo Gr,	2240	−2.5	−12.9	17.6	17.6	0.1
HT-18	Bright purple cherty Dol.	Daguanshan Fm., Hutuo Gr.	2200	0.5	−9.5	21.0	19.4	0.5
Changcheng and Jixian Systems in the Jixian profile								
JX-40-7	Black siliceous bands	Wumishan Fm. (top), Jixian System	1230	0.9	−7.2	23.4	25.5	2.7
JX-40-6	Black siliceous bands	Wumishan Fm. (top), Jixian System	1230	0.7	−9.4	21.2	27.2	2.8
JX-37-4	Black siliceous bands	Wumishan Fm. (top), Jixian System	1240	1.0	−11.1	19.4	26.0	2.1
JX-37-3	Black siliceous bands	Wumishan Fm. (top), Jixian System	1240	0.7	−9.3	21.2	25.9	2.7
JX-37-2	Black siliceous bands	Wumishan Fm. (top), Jixian System	1240	1.0	−9.0	21.6	26.0	2.6
JX-37-1	Black siliceous bands	Wumishan Fm. (top), Jixian System	1240	0.7	−9.2	21.3	26.7	2.4
JX-36-12	Siliceous bands in dolomite	2^nd^ member, Wumishan Fm., Jixian System	1250	−0.3	−10.8	19.7	25.3	2.3
JX-36-11	Siliceous bands in dolomite	2^nd^ member, Wumishan Fm., Jixian System	1250	−0.4	−8.4	22.2	25.3	2.4
JX-36-10	Siliceous bands in dolomite	2^nd^ member, Wumishan Fm., Jixian System	1250	−0.7	−9.3	21.3	25.8	2.4
JX-36-9	Siliceous bands in dolomite	2^nd^ member, Wumishan Fm., Jixian System	1250	−0.5	−7.7	22.9	26.6	2.2
JX-36-7	Siliceous bands in dolomite	2^nd^ member, Wumishan Fm., Jixian System	1250	−0.5	−6.2	24.5	26.0	2.3
JX-36-6	Siliceous bands in dolomite	2^nd^ member, Wumishan Fm., Jixian System	1250	−0.7	−6.4	24.2	26.4	2.4
JX-36-5	Siliceous bands in dolomite	2^nd^ member, Wumishan Fm., Jixian System	1250	−0.7	−6.3	24.4	26.0	2.4
JX-36-3	Siliceous bands in dolomite	2^nd^ member, Wumishan Fm., Jixian System	1250	−0.7	−7.3	23.4	25.8	2.6
JX-36-1	Siliceous bands in dolomite	2^nd^ member, Wumishan Fm., Jixian System	1250	−0.8	−10.6	20.0	25.4	2.2
JX-34	Siliceous bands in dolomite	2^nd^ member, Wumishan Fm., Jixian System	1270	−1.1	−5.9	24.8	25.1	2.2
JX-33-12	Siliceous bands in dolomite	2^nd^ member, Wumishan Fm., Jixian System	1280	−1.2	−11.1	19.5	25.8	2.2
JX-33-11	Siliceous bands in dolomite	2^nd^ member, Wumishan Fm., Jixian System	1280	−1.0	−9.6	20.9	26.3	2.5
JX-33-10	Siliceous bands in dolomite	2^nd^ member, Wumishan Fm., Jixian System	1280	−1.0	−11.0	19.6	25.3	2.4
JX-33-8	Siliceous bands in dolomite	2^nd^ member, Wumishan Fm., Jixian System	1280	−1.2	−9.4	21.2	25.1	2.3
JX-33-7	Siliceous bands in dolomite	2^nd^ member, Wumishan Fm., Jixian System	1280	−1.2	−11.8	18.7	24.7	2.4
JX-33-6	Siliceous bands in dolomite	2^nd^ member, Wumishan Fm., Jixian System	1280	−1.1	−8.9	21.6	24.6	2.3
JX-33-5	Siliceous bands in dolomite	2^nd^ member, Wumishan Fm., Jixian System	1280	−1.1	−8.8	21.8	25.2	2.2
JX-33-3	Siliceous bands in dolomite	2^nd^ member, Wumishan Fm., Jixian System	1280	−1.0	−8.1	22.5	26.1	2.3
JX-32	Siliceous bands in dolomite	2^nd^ member, Wumishan Fm., Jixian System	1290	−1.2	−6.7	23.9	24.6	2.1
JX-27-6	Siliceous bands in dolomite	2^nd^ member, Wumishan Fm., Jixian System	1325	−0.6	−6.5	24.1	26.8	3.2
JX-27-5	Siliceous bands in dolomite	2^nd^ member, Wumishan Fm., Jixian System	1325	−0.8	−7.6	23.1	27.3	3.5
JX-27-4	Siliceous bands in dolomite	2^nd^ member, Wumishan Fm., Jixian System	1325	−0.7	−6.6	24.1	27.8	3.1
JX-27-3	Siliceous bands in dolomite	2^nd^ member, Wumishan Fm., Jixian System	1325	−0.6	−6.1	24.6	27.7	3.2
JX-27-2	Siliceous bands in dolomite	2^nd^ member, Wumishan Fm., Jixian System	1325	−0.1	−7.5	23.2	27.6	3.6
JX-26-6	Siliceous bands in dolomite	2^nd^ member, Wumishan Fm., Jixian System	1335	−0.9	−5.9	24.8	25.3	3.9
JX-26-4	Siliceous bands in dolomite	2^nd^ member, Wumishan Fm., Jixian System	1335	−0.3	−5.9	24.7	25.2	3.9
JX-26-3	Siliceous bands in dolomite	2^nd^ member, Wumishan Fm., Jixian System	1335	−0.2	−6.0	24.7	24.2	3.1
JX-26-2	Siliceous bands in dolomite	2^nd^ member, Wumishan Fm., Jixian System	1335	−0.2	−6.2	24.5	23.1	2.3
JX-25	Siliceous bands in dolomite	2^nd^ member, Wumishan Fm., Jixian System	1345	−0.7	−7.1	23.5	25.2	2.7
JX-20-8	Siliceous bands in dolomite (with bitumen)	Yangzhuang Fm., Jixian System	1400	−0.8	−8.6	22.0	21.7	2.6
JX-20-7	Siliceous bands in dolomite (with bitumen)	Yangzhuang Fm., Jixian System	1400	−1.3	−7.6	23.0	23.3	3.1
JX-20-6	Siliceous bands in dolomite (with bitumen)	Yangzhuang Fm., Jixian System	1400	−0.8	−8.5	22.1	22.1	2.7
JX-20-4	Siliceous bands in dolomite (with bitumen)	Yangzhuang Fm., Jixian System	1400	−0.6	−7.6	23.0	22.9	2.5
JX-20-2	Siliceous bands in dolomite (with bitumen)	Yangzhuang Fm., Jixian System	1400	−1.4	−8.8	21.8	23.1	2.8
JX-19	Siliceous bands in dolomite	4^th^ member, Gaoyuzhuang Fm., Changcheng System	1410	−0.5	−6.0	24.7	25.9	1.8
JX-10	Siliceous bands in dolomite	2^nd^ member, Gaoyuzhuang Fm., Changcheng System	1520	−0.6	−3.5	27.2	27.4	1.0
JX-09	Black siliceous bands	2^nd^ member, Gaoyuzhuang Fm., Changcheng System	1540	−0.7	−6.9	23.7	27.7	0.6
JX-05	Siliceous bands in dolomite	2^nd^ member, Gaoyuzhuang Fm., Changcheng System	1580	0.5	−4.9	25.8	23.5	1.4
JX-04	Siliceous stromatolite	Gaoyuzhuang Fm. (B), Changcheng System	1600	0.3	−6.9	23.7	24.2	1.1
JX-75	Dike of siliceous stromatolite	Dahongyu Fm., Changcheng System	1610				22.4	0.6
JX-74	Siliceous stromatolites	Dahongyu Fm., Changcheng System	1620				25.8	0.5
JX-73	Siliceous stromatolites	Dahongyu Fm., Changcheng System	1625				24.7	0.7
JX-71	Laminated chert	Dahongyu Fm., Changcheng System	1628				25.2	0.9
JX-70	Siliceous band	Dahongyu Fm., Changcheng System	1630				25.1	0.5
Changcheng and Jixian Systems in the Shisanlin profile								
SSL-07	Chert band in Dol	3^rd^ Member, Gaoyuzhuang Fm., Changcheng System	1550	0.2	−7.5	23.1	16.5	1.9
SSL-08	Chert band	3^rd^ Member, Gaoyuzhuang Fm., Changcheng System	1530	0	−6.7	24.0	25.2	2.4
SSL-09	Chert band	3^rd^ Member, Gaoyuzhuang Fm., Changcheng System	1510	−1.0	−4.8	25.9	17.6	2.3
SSL-10	Siliceous band in stromatolite	3^rd^ Member, Gaoyuzhuang Fm., Changcheng System	1490	−0.5	−6.4	24.3	16.2	2.6
SSL-11	Siliceous nodule in Dol	4^th^ Member, Gaoyuzhuang Fm., Changcheng System	1480	−0.9	−4.3	26.4	24.7	1.8
SSL-12	Siliceous band in Dol	4^th^ Member, Gaoyuzhuang Fm., Changcheng System	1470	−0.8	−4.5	26.2	22.1	2.0
SSL-13	Siliceous nodule in Dol	4^th^ Member, Gaoyuzhuang Fm., Changcheng System	1460	−0.8	−4.9	25.8	17.4	1.9
SSL-14	Milk chert band	4^th^ Member, Gaoyuzhuang Fm., Changcheng System	1450	0.1	−4.2	26.5	17.4	1.7
SSL-15	Siliceous nodule	4^th^ Member, Gaoyuzhuang Fm., Changcheng System	1450	−0.5	−5.9	24.8	20.6	1.2
SSL-16	Chert band	4^th^ Member, Gaoyuzhuang Fm., Changcheng System	1440	−0.3	−5.3	25.4	17.6	1.2
SSL-18	Siliceous stromatolite	4^th^ Member, Gaoyuzhuang Fm., Changcheng System	1410	−0.4	−4.4	26.3	24.0	2.4
SSL-19	Red chert rocks in Dol	Yangzhuang Fm., Jixian System	1400	−0.7	−6.7	24.1	24.6	2.4
SSL-20	Green chert band in Dol	Yangzhuang Fm., Jixian System	1390	−1.4	−3.6	27.2	25.0	2.4
SSL-21	Grey siliceous nodule	Yangzhuang Fm., Jixian System	1380	−2.3	−3.7	27.0	24.5	2.7
SSL-22	Massive chert	1^st^ Member, Wumishan Fm., Jixian System	1375	−0.1	−4.7	26.0	23.0	1.6
SSL-23	Chert lenses	2^nd^ Member, Wumishan Fm., Jixian System	1355	−0.2	−5.0	25.7	20.9	2.7
SSL-24	Banded chert rocks	2^nd^ Member, Wumishan Fm., Jixian System	1330	−0.3	−5.8	24.9	24.3	2.2
SSL-25	Fine banded chert rocks	2^nd^ Member, Wumishan Fm., Jixian System	1320	−0.6	−5.3	25.4	22.5	1.9
SSL-26	White-grey chert rocks	2^nd^ Member, Wumishan Fm., Jixian System	1310	−0.9	−7.2	23.4	25.8	2.1
SSL-27	Silica-bearing brecciate Dol	3^rd^ Member, Wumishan Fm., Jixian System	1300	0.4	−5.6	25.1	22.6	2.5
SSL-28	Siliceous nodule in Dol	3^rd^ Member, Wumishan Fm., Jixian System	1290	0.6	−5.2	25.5	19.3	2.1
SSL-29	White-grey quartzite	3^rd^ Member, Wumishan Fm., Jixian System	1280	−0.9	−3.9	26.8	20.6	2.6
SSL-30	Black-grey siliceous Dol	3^rd^ Member, Wumishan Fm., Jixian System	1270	−1.1	−4.8	25.9	16.9	2.2
SSL-31	Black-grey siliceous nodule	3^rd^ Member, Wumishan Fm., Jixian System	1260	−0.8	−4.3	26.4	16.6	2.0
SSL-33	Black-grey siliceous band	4^th^ Member, Wumishan Fm., Jixian System	1250	−0.1	−4.6	26.1	14.1	1.1
SSL-34	Siliceous stromatolite	4^th^ Member, Wumishan Fm., Jixian System	1240	−0.2	−4.8	25.9	25.5	3.4
SSL-35	Siliceous nodule in white-grey Dol	4^th^ Member, Wumishan Fm., Jixian System	1230	0.3	−4.1	26.6	15.7	1.8
SSL-36	Black chert band in white-grey Dol	4^th^ Member, Wumishan Fm., Jixian System	1220	0.2	−3.8	26.9	16.1	1.7
SSL-37	Chert band in grey Dol	4^th^ Member, Wumishan Fm., Jixian System	1200	1.2	−5.4	25.3	17.4	1.7

Analytic precisions are ± 0.1‰ (2σ) and ± 0.2‰ (2σ) for the δ^30^Si_NBS-28_ and δ^18^O_V-SMOW_ of cherts, respectively.

Analytic precisions are ± 0.2‰ (2σ) for the δ^13^C_V-PDB_, δ^18^O_V-PDB_ and δ^18^O_V-SMOW_ of dolomites, respectively.
